# Enhanced visible-light photodegradation of fluoroquinolone-based antibiotics and *E. coli* growth inhibition using Ag–TiO_2_ nanoparticles†

**DOI:** 10.1039/d0ra10403e

**Published:** 2021-04-13

**Authors:** Jiao Wang, Ladislav Svoboda, Zuzana Němečková, Massimo Sgarzi, Jiří Henych, Nadia Licciardello, Gianaurelio Cuniberti

**Affiliations:** Institute for Materials Science, Max Bergmann Centre of Biomaterials, Dresden Center for Nanoanalysis, TU Dresden 01062 Dresden Germany nadia.licciardello@tu-dresden.de nadia.licciardello@inl.int gianaurelio.cuniberti@tu-dresden.de; IT4Innovations, VŠB – Technical University of Ostrava 17. listopadu 15/2172 708 33 Ostrava Czech Republic; Nanotechnology Centre, CEET, VSB-Technical University of Ostrava 17. listopadu 15/2172 708 33 Ostrava Czech Republic; Institute of Inorganic Chemistry, Czech Academy of Sciences Husinec-Řež 1001 250 68 Řež Czech Republic

## Abstract

Antibiotics in wastewater represent a growing and worrying menace for environmental and human health fostering the spread of antimicrobial resistance. Titanium dioxide (TiO_2_) is a well-studied and well-performing photocatalyst for wastewater treatment. However, it presents drawbacks linked with the high energy needed for its activation and the fast electron–hole pair recombination. In this work, TiO_2_ nanoparticles were decorated with Ag nanoparticles by a facile photochemical reduction method to obtain an increased photocatalytic response under visible light. Although similar materials have been reported, we advanced this field by performing a study of the photocatalytic mechanism for Ag–TiO_2_ nanoparticles (Ag–TiO_2_ NPs) under visible light taking in consideration also the rutile phase of the TiO_2_ nanoparticles. Moreover, we examined the Ag–TiO_2_ NPs photocatalytic performance against two antibiotics from the same family. The obtained Ag–TiO_2_ NPs were fully characterised. The results showed that Ag NPs (average size: 23.9 ± 18.3 nm) were homogeneously dispersed on the TiO_2_ surface and the photo-response of the Ag–TiO_2_ NPs was greatly enhanced in the visible light region when compared to TiO_2_ P25. Hence, the obtained Ag–TiO_2_ NPs showed excellent photocatalytic degradation efficiency towards the two fluoroquinolone-based antibiotics ciprofloxacin (92%) and norfloxacin (94%) after 240 min of visible light irradiation, demonstrating a possible application of these particles in wastewater treatment. In addition, it was also proved that, after five Ag–TiO_2_ NPs re-utilisations in consecutive ciprofloxacin photodegradation reactions, only a photocatalytic efficiency drop of 8% was observed. Scavengers experiments demonstrated that the photocatalytic mechanism of ciprofloxacin degradation in the presence of Ag–TiO_2_ NPs is mainly driven by holes and ˙OH radicals, and that the rutile phase in the system plays a crucial role. Finally, Ag–TiO_2_ NPs showed also antibacterial activity towards *Escherichia coli* (*E. coli*) opening the avenue for a possible use of this material in hospital wastewater treatment.

## Abstract 2

Ag nanoparticles decorated-TiO_2_ P25 are a viable alternative for the degradation, through a rutile-mediated mechanism, of fluoroquinolone-based antibiotics under visible light irradiation and, at the same time, for bacteria inactivation in water.

## Graphical abstract

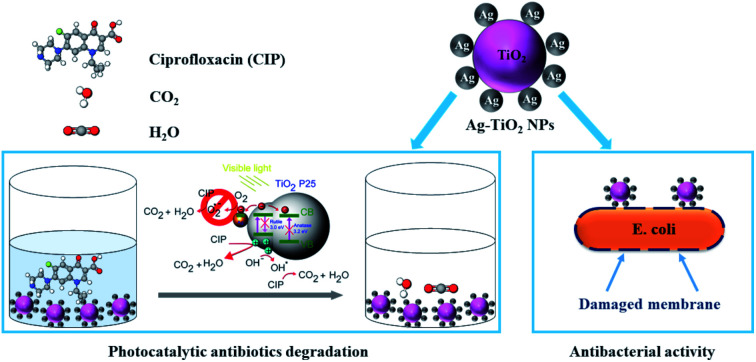

## Introduction

Water is essential for life. However, the amount of fresh, accessible water on Earth is very limited. With the increasing industrial production and the growth of the world's population, water pollution has become a severe issue that affects human and environmental health.^1–4^ In the last years, the presence of active pharmaceutical ingredients (API) in water has attracted increasing attention due to the several damaging effects which they can cause.^5–8^ API are “pseudo-persistent” pollutants because their transformation/elimination rate is balanced by their continuous input into the environment.^7,9^ Antibiotics are a widely studied type of API because they are one of the causes of the development of multiple resistant bacteria and because they are detected frequently in the aquatic environment.^10,11^ The reported levels of antibiotics in wastewater are in the range of ng L^−1^ to μg L^−1^. However, the concentration of wastewater discharged by pharmaceutical factories in some developing countries has reached mg L^−1^.^12,13^ Therefore, the elimination of antibiotics is an emergent problem to be solved. For example, ciprofloxacin (CIP) and norfloxacin (NFX), both belonging to the fluoroquinolone family, have become common antibiotics in wastewater from wastewater treatment plants.^14^ Being widely present in wastewater, they can threaten the ecological environment and human health by inducing the increase of bacterial drug resistance.^15^ Several techniques have been used to remove antibiotics, such as biological treatments,^16^ membrane processes,^17^ chemical and electrochemical techniques,^18,19^ and adsorption procedures.^20^ Nevertheless, most of these techniques are complex or require large spaces and, mostly, they are not able to fully eliminate small traces of antibiotics in wastewater. Advanced oxidation processes (AOPs), and especially those involving semiconductor photocatalysts, have attracted extensive attention.^4,21,22^ AOPs are processes in which highly oxidising species, like hydroxyl radicals and other reactive oxygen species, are produced and can degrade target pollutants in wastewater.^23^ Among AOPs, photocatalysis is one of the most studied processes since it usually allows the elimination of traces of pollutants in water and, ideally, their full mineralisation.

Among semiconductor materials, TiO_2_ is one of the most used photocatalysts because of its large specific surface area, relatively low toxicity, low cost and high catalytic activity.^24^ In 1972, Fujishima and Honda were the first to report the photocatalytic activity of TiO_2_, which was used as the photo-anode in UV-light induced electrochemical water splitting.^25^ Ever since, this material has been widely explored in photocatalysis for several applications, including wastewater treatment. However, TiO_2_, especially in its more commonly used crystalline phase anatase, has a wide bandgap (∼3.2 eV), which requires UV light irradiation for the photo-activation. UV light contribution in solar irradiance spectrum is limited (5%) compared to visible (43%) and infrared light (52%), which restricts TiO_2_ actual environmental application under visible or solar light irradiation.^26^

Doping TiO_2_ with metal or non-metal atoms or modifying the surface of TiO_2_ with noble metals are some of the suggested solutions to overcome this problem.^27–31^ Noble metals play an important role in the visible-light-activated photocatalytic process^24,32^ by lowering the bandgap, promoting electron–hole pairs separation and/or introducing additional catalytically active sites on the surface of TiO_2_. Among them, Ag is a promising candidate, owing to its relatively low cost (compared with gold for instance), easy preparation and particularly high performance in enhancing the TiO_2_ photocatalytic activity under visible light.^33^ When the TiO_2_ surface is decorated with Ag NPs, the photocatalytic response under visible light is enhanced because of the localised surface plasmon resonance effect of Ag NPs and the increased separation efficiency of photogenerated electron–hole pair, since Ag can act as an electron acceptor.^4,34^ Besides, Ag NPs have a long-time known strong antibacterial activity.^4^

However, a deep understanding of the photocatalytic mechanism for Ag–TiO_2_ NPs under visible light irradiation is often missing in the literature^35,36^ and authors usually ignored the role played by the rutile phase of TiO_2_ when using commercial TiO_2_ P25.^37^

In this manuscript, we report on the surface modification of the commercial Evonik Aeroxide® TiO_2_ P25 with Ag nanoparticles using a facile photochemical reduction method.^38^ The phase composition and microstructure as well as the optical properties of the as-obtained Ag–TiO_2_ NPs were analysed. The photocatalytic performance under visible light irradiation for the photodegradation of the fluoroquinolone-based antibiotics CIP and NFX was explored. The possibility to reuse the same batch of Ag–TiO_2_ NPs was examined. As a model, the photocatalytic mechanism underlying the degradation of CIP in the presence of Ag–TiO_2_ NPs was investigated shedding some light on it, and taking into consideration the role played by the rutile phase. Moreover, qualitative experiments on the antibacterial activity of the Ag–TiO_2_ NPs against *E. coli* were also performed, showing antimicrobial ability and demonstrating the potential disinfection properties of these particles in addition to their photocatalytic activity. These findings not only contribute to better explain the mechanism behind the photocatalytic activity of Ag–TiO_2_ NPs under visible light, but envisage the prospective future use of these particles, for instance, in hospital wastewater treatment, where both bacteria and pharmaceuticals might be present.

## Experimental section

### Materials

Titanium dioxide Aeroxide® TiO_2_ P25 was purchased from Evonik. Silver nitrate (AgNO_3_, ≥99.0%), hydrochloric acid (HCl, 37%), ciprofloxacin (CIP, ≥98.0%), norfloxacin (NFX, ≥98.0%), methanol (CH_3_OH, ≥99.9%), ethylenediaminetetraacetic acid (EDTA, ≥99.0%), isopropanol (≥99.5%) and Luria-Bertani (LB) broth were purchased from Sigma-Aldrich. Agar–agar was purchased from Merck. The strain of *Escherichia coli* used was the following: *E. coli* YFP, MG1655 galK::SYFP2-FRT. The ultrapure water was produced by a MembraPure Astacus system (MembraPure GmbH, Hennigsdorf, Germany).

### Preparation of Ag–TiO_2_ NPs

Ag–TiO_2_ NPs were prepared by a photochemical reduction method.^38^ TiO_2_ P25 (0.2 g) was dispersed in AgNO_3_ solution (0.2 mol L^−1^, 50 mL) at room temperature. After 24 h stirring, 40 mL methanol were added. Subsequently, the system was irradiated with a 10 W LED chip (*λ*_max_ = 365 nm) for 30 min to reduce the Ag^+^ ions to metallic Ag nanoparticles. Finally, the as-prepared samples were washed with ultrapure water and dried at 70 °C for 24 h.

### Characterisation of Ag–TiO_2_ NPs

The morphology and size of Ag–TiO_2_ NPs were individually examined by high-resolution transmission electron microscopy (HR-TEM). HR-TEM images were acquired using a 200 kV TEM microscope FEI Talos F200X, which combines high-resolution S/TEM with energy-dispersive X-ray spectroscopy (EDS) signal detection, and 3D chemical characterisation with compositional mapping. As specimen support for TEM investigations, a microscopic copper grid covered by a thin transparent holey carbon film was used. Ag–TiO_2_ NPs were ultrasonicated for few minutes, and 3 μL of this solution were dropped on the grid and evaporated at room temperature. The grid with samples was cleaned by UV light irradiation under vacuum just before the measurement. All measured images were processed by ImageJ software (National Institute of Health, USA). The particle-size distribution of Ag NPs was statistically evaluated by using Origin software (Northampton, USA). The diffraction patterns of Ag–TiO_2_ NPs were evaluated using Process Diffraction software (János L. Lábár, Hungary).

The chemical composition of Ag–TiO_2_ NPs was examined *via* Attenuated Total Reflectance-Fourier Transform Infrared (ATR-FTIR) spectroscopy. ATR-FTIR spectra were recorded on IRAffinity-1S (Shimadzu, Japan) equipped with GladiATR-10 as ATR accessory. The spectra were acquired in transmission mode, acquiring 32 scans with a resolution of 2 cm^−1^ in the wavenumber range from 4000 to 500 cm^−1^.

The crystalline structure of the prepared Ag–TiO_2_ NPs was investigated by X-ray powder diffraction (XRD). XRD patterns were obtained by Bruker D8 Advance diffractometer with *λ*_Cu_ = 0.15406 nm. Prepared powder samples were placed into a rotational holder.

The measurement of optical absorption and bandgap energies was obtained by diffuse reflectance UV-Vis (UV-Vis DRS) spectra in the range of 280–800 nm with a Shimadzu spectrophotometer UV-2600. The obtained spectra were measured in diffuse reflectance mode and expressed in terms of absorbance by means of Kubelka–Munk function.

The Fluorometer FLS920 (Edinburgh Instrument Ltd, UK), equipped with a 450 W xenon lamp, was used for the acquisition of photoluminescence spectra. The excitation wavelength 325 nm was used for all the measurements. All the samples were measured in powdered form.

Specific surface area (*A*_BET_) was measured with the surface area analyser SA9601 (HORIBA Scientific, Japan). The dry powdered samples were degassed for 6 hours at 80 °C, and subsequently the six-point analysis was applied.

### Photocatalytic activity measurements

CIP and NFX concentrations were measured *via* UV-Vis spectrophotometer (Cary 100 UV-Vis) with quartz cuvettes.

The photocatalytic activity of TiO_2_ P25 and Ag–TiO_2_ NPs catalysts was evaluated by the chemical degradation of CIP and NFX antibiotics. In detail, 15 mg of catalyst were added to 50 mL of antibiotic solution (CIP or NFX) to reach a catalyst concentration of 300 mg L^−1^. The antibiotic solution had an initial concentration of 3 mg L^−1^ (pH = 3, adjusted by using 1 M HCl). The selection of pH = 3 for the CIP and NFX solutions was performed to ensure better solubility of the antibiotics in water. According to the literature, indeed, the CIP and NFX have two acid dissociation constant (p*K*_a_) values of 5.9 and 8.9 for CIP, and 6.4 and 8.7 for NFX. Therefore, they are positively charged at pH = 3,^39^ which increases their solubility in water.

As visible light source, an artificial lamp was used (Ingenieurbüro Mencke & Tegtmeyer GmbH, Germany) with Susicontrol software (version 2.9.0) to monitor the light intensity by a silicon irradiance sensor. As reported by the producer, the visible light spectrum is similar to the natural solar light, but with negligible UV light content (wavelengths ranging from 400 to 1100 nm). The irradiance used during the experiments was 98 W m^−2^. The distance between the visible light source and the surface of the CIP or NFX solution was 9.4 cm. The catalyst particles were added into the CIP or NFX solution under stirring. The mixture was kept in dark conditions for 30 min to establish the adsorption–desorption equilibrium before irradiation with visible light. The absorbance changes for CIP and NFX were monitored at 277 nm (for CIP) or 278 nm (for NFX) at different times of irradiation by using a UV-Vis spectrophotometer. During the process, samples were withdrawn at times 0 min (before irradiation) and after 5 min, 10 min, 20 min, 30 min, 50 min, 70 min, 90 min, 120 min, 150 min, 180 min, 210 min, 240 min of visible light irradiation and centrifuged for 20 min at 20 817*g* and 20 °C, in order to remove the suspended nanoparticles.

A similar procedure was followed for the recycling experiments: after each CIP photocatalytic degradation, the Ag–TiO_2_ NPs were recovered by centrifugation (10 min at 20 817*g*), washed once with 0.1 M HCl and twice with ultrapure water, dried at 40 °C in the oven and reused for the subsequent photocatalytic degradation of fresh CIP solution.

Besides, in order to study the photocatalytic degradation mechanism of CIP by Ag–TiO_2_ NPs, different scavengers, namely EDTA (17 mg), isopropanol (5 mL) and AgNO_3_ (17 mg), were introduced into the system (total volume 50 mL) as holes (h^+^) scavenger, ˙OH radicals scavenger, and electrons (e^−^) scavenger, respectively.

### Antibacterial tests

All the laboratory supplies were sterilised by autoclaving at 121 °C for 20 min. All the experiments were performed under sterile conditions.

The *E. coli* suspension culture was prepared as follows. Firstly, 10 g of LB broth was added to 500 mL of ultrapure water in a sterile flask under stirring for 15 min. Secondly, the LB broth was sterilised in the autoclave and subsequently cooled to room temperature. Subsequently, 50 mL of LB broth were transferred to a new sterile flask to which 2 mL of the *E. coli* culture were added while working in the laminar flow chamber. Finally, this solution was incubated overnight under continuous shaking at 37 °C.

In order to prepare the LB agar plate, 10 g of LB broth, 7.5 g of Agar–agar were added to 500 mL of ultrapure water in a sterile flask under stirring for 15 min. The medium was afterwards sterilised in the autoclave and subsequently cooled down. Finally, a sufficient volume of the LB-agar was transferred into a sterile Petri dish while working in the laminar flow chamber. The as-prepared LB agar plate was stored at 4 °C for future use.

The antibacterial activity of Ag–TiO_2_ NPs against *E. coli* was evaluated by a modified streaking method.^40,41^ The specific experimental steps are as follows. The LB agar plate was divided into two parts by using an antibacterial stick. Five streaks were made for each part. A solution of *E. coli* in LB broth (20 μL, 10^8^ cells per mL) was placed in each streak on the left part of the plate. Ag–TiO_2_ NPs (0.2 mg) and *E. coli* in LB broth (20 μL, 10^8^ cells per mL) were placed in each streak on the right part of the plate. The obtained plate was incubated in dark conditions at 37 °C for 24 h, and examined for testing the strain inhibition in the presence of Ag–TiO_2_ NPs. As a comparison, another LB agar plate was divided into two parts by using the antibacterial stick. Five streaks were made for each part. *E. coli* bacteria in LB broth (20 μL, 10^8^ cells per mL) were placed in each streak on the left part of the plate. TiO_2_ P25 (0.2 mg) and *E. coli* in LB broth (20 μL, 10^8^ cells per mL) were placed in each streak on the right part of the plate. The obtained plate was incubated in dark conditions at 37 °C for 24 h, and examined for testing the strain inhibition in the presence of TiO_2_ P25.

## Results and discussion

### Characterisation of Ag–TiO_2_ NPs

**Scheme 1:**
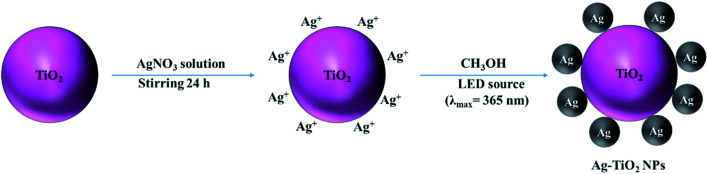
Synthetic route for the preparation of Ag–TiO_2_ NPs.

The synthetic path for the preparation of Ag–TiO_2_ NPs is illustrated in Scheme 1. Briefly, Ag–TiO_2_ NPs were prepared using a photochemical reduction method.^38^ The Ag NPs were formed on the surface of the commercial TiO_2_ NPs through photochemical reduction of AgNO_3_ under 365 nm LED irradiation.

**Fig. 1:**
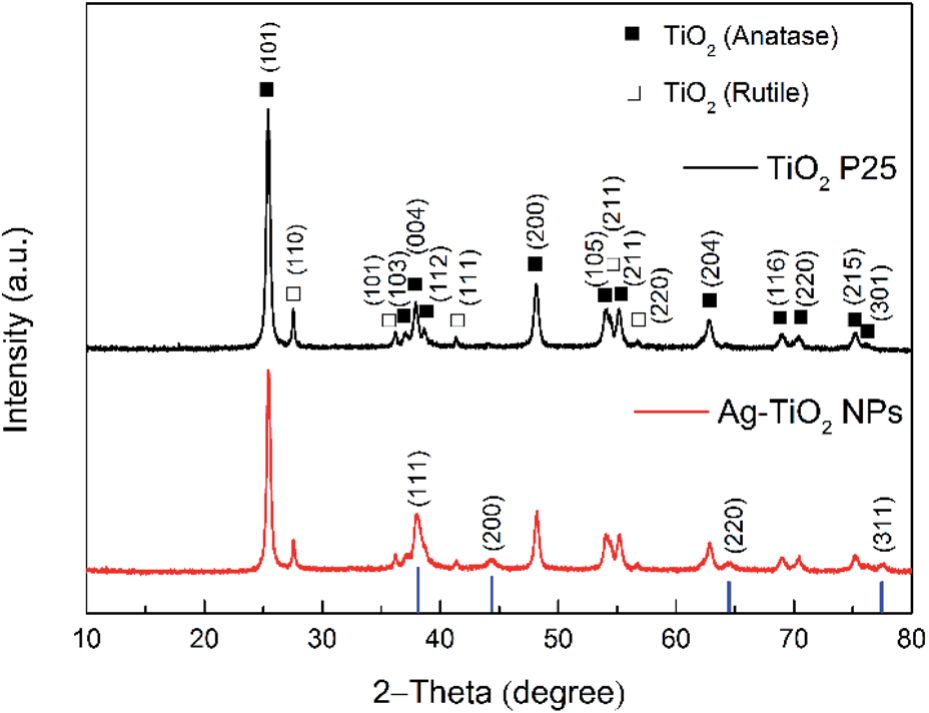
Powder XRD patterns of TiO_2_ P25 and Ag–TiO_2_ NPs. The blue lines represent the diffraction peaks positions for the typical Ag XRD pattern (JCPDS no. 87-0717).

The ATR-FTIR spectra of TiO_2_ P25 and Ag–TiO_2_ NPs are shown in Fig. S1 (ESI†) and confirm that the Ag NPs did not modify the chemical structure of TiO_2_ P25. However, the concentration of Ag NPs compared with TiO_2_ NPs in the sample is very low and it is therefore difficult to confirm their presence by FTIR spectroscopy. In order to prove the presence of Ag NPs in the Ag–TiO_2_ NPs system, the sample was thus characterised by XRD. The XRD patterns of TiO_2_ P25 and Ag–TiO_2_ NPs are shown in Fig. 1.

**Fig. 2:**
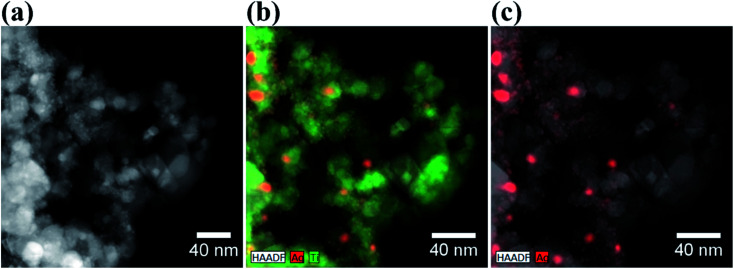
(a) HRTEM image of Ag–TiO_2_ NPs; (b) EDS map of Ag–TiO_2_ NPs with Ag (red) and Ti (green) atomic distribution; (c) EDS map of Ag–TiO_2_ NPs with Ag atomic distribution (in red).

The XRD pattern for the TiO_2_ P25 shows several typical peaks for both anatase and rutile, as expected. Besides the diffraction pattern similar to the one of TiO_2_ P25, the Ag–TiO_2_ NPs exhibit four additional main diffraction peaks at 38.116°, 44.277°, 64.426° and 77.472°. These peaks correspond to the (111), (200), (220) and (311) planes of Ag (JCPDS no. 87-0717; the blue bars in Fig. 1).^42^ These results prove that the obtained Ag–TiO_2_ NPs contain Ag. Furthermore, the estimated weight percentages of anatase, rutile and silver based on the XRD patterns are 82.12%, 12.76% and 5.12% respectively. The morphology and structure of Ag–TiO_2_ NPs were characterised by HRTEM measurements. Fig. 2(a) shows the HRTEM image of Ag–TiO_2_ NPs, where the Ag NPs are observed as darker and smaller dots on the TiO_2_ NPs surface. As it is shown in Fig. 2(a), the Ag NPs are very finely and homogeneously dispersed on the TiO_2_ surface.

The size distribution of Ag NPs is shown in Fig. S2.† The average size of the Ag NPs is 23.9 ± 18.3 nm. However, these represent probably the agglomerated primary Ag NPs present in higher amount and more easily individuated by HRTEM. From the size distribution graph in Fig. S2(b),† it is clear that the primary Ag NPs size lies between 10 and 20 nm, although also smaller particles are visible in the STEM-HAADF micrograph in Fig. S2(a).† Some Ag NPs agglomerates with size bigger than 100 nm are also present. The EDS elemental maps are shown in Fig. 2(b) and (c). From these maps, it is even more evident that Ag NPs are localised mainly at the surface of the TiO_2_ NPs. The successfully Ag modification on the surface of TiO_2_ was confirmed by EDS analysis, where the weight percentage of Ag is about 5%, which is a similar result to the one obtained by XRD patterns.

**Fig. 3:**
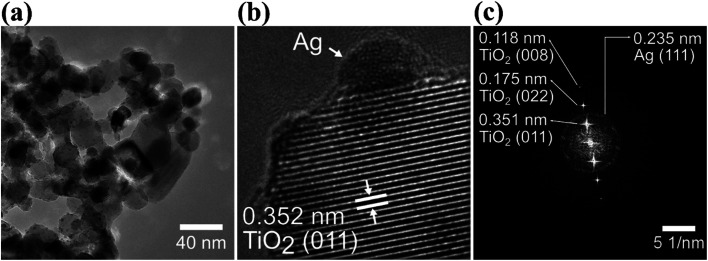
HRTEM images at low magnification (a) and high magnification (b) of Ag–TiO_2_ NPs; (c) SAED (selected area electron diffraction) pattern of Ag–TiO_2_ NPs.

Fig. 3(a) and (b) clearly show that Ag NPs are evenly distributed on the surface of TiO_2_. The SAED image in Fig. 3(c) shows the crystalline lattice of TiO_2_ NPs displaying the lattice spacing of 0.351 nm, 0.175 nm and 0.118 nm, which are characteristics for the crystalline planes (011), (022) and (008), respectively. Besides, in the SAED image, it is also possible to individuate the lattice spacing of 0.235 nm for the (111) planes of Ag NPs.

**Fig. 4:**
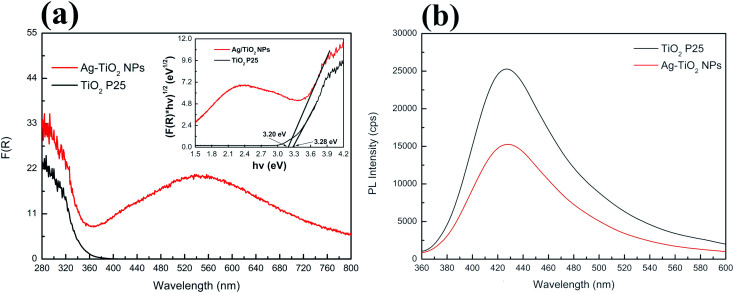
(a) UV-Vis absorption spectra of TiO_2_ P25 and Ag–TiO_2_ NPs with the inset of Tauc plot and (b) PL spectra of TiO_2_ P25 and Ag–TiO_2_ NPs.

UV-Vis diffuse reflectance spectroscopy was used to investigate optical absorption and bandgap energy of both TiO_2_ P25 and Ag–TiO_2_ NPs. Fig. 4(a) shows the DRS spectra of pure TiO_2_ P25 and Ag–TiO_2_. Both materials show the typical absorption edge in the UV region around 380 nm. However, in the case of Ag–TiO_2_ NPs, the spectrum contains a broad band in the visible region ranging from 385 to 800 nm with the maximum absorbance in the range between 530 and 550 nm, that is ascribable to the localised surface plasmon resonance (LSPR) of Ag particles.^43^ The position of the maximum absorbance around 540 nm could be influenced by the used Ag precursor in the Ag–TiO_2_ NPs synthesis. Albiter *et al.*^44^ studied the synthesis of Ag–TiO_2_ NPs in the presence of different Ag precursors, and they found out that the maximum absorbance in the visible light region strongly depended on the chosen salt for synthesis. Moreover, the position of the maximum absorbance is influenced by particle size, particles distribution on the surface of TiO_2_ and chemical interaction between Ag NPs and TiO_2_.^44^ The DRS spectra also represent an indirect proof of the TiO_2_ surface coverage with Ag NPs, since the obtained spectra resemble the plasmon surface absorption typical of Ag NPs.^45^ Similar effects, caused by TiO_2_ NPs' surface decoration with Ag NPs, on the absorption properties of the final system were already observed in other reported Ag–TiO_2_ NPs samples.^35^

Furthermore, Ag NPs at the interface with TiO_2_ slightly shifted the electronic bandgap of TiO_2_ to higher wavelength due to the creation of a metal–semiconductor junction (Schottky contact). The estimated bandgap energy for TiO_2_ P25 is 3.28 eV, corresponding to an absorption edge around 378 nm, while in the case of Ag–TiO_2_ NPs, the estimated bandgap energy is around 3.2 eV (387.5 nm). Thus, the surface decoration of TiO_2_ with Ag NPs slightly affected the bandgap energy of TiO_2_. However, it is evident that the photo-response of the Ag–TiO_2_ NPs is greatly enhanced in the visible light region when compared to bare TiO_2_.

To investigate the promoted electron–hole pairs separation and the recombination of these photo-excited carriers, the emission spectra of the samples were acquired (*λ*_exc_ = 325 nm, Fig. 4(b)). A lower emission intensity is desirable as it might indicate a lower probability of electron–hole recombination and an enhanced longer lifetime of the photogenerated charge carriers resulting in higher photocatalytic activity. As shown in Fig. 4(b), both TiO_2_ P25 and Ag–TiO_2_ NPs show the maximum photoluminescence (PL) emission intensity around 428 nm, but the intensity is lowered by 40% for Ag–TiO_2_ NPs demonstrating a lower electron–hole recombination rate in this material in respect to TiO_2_ P25. Similar PL results are reported for Ag/TiO_2_ (ref. 46) and Ag_2_O/TiO_2_ (ref. 47) systems.

Finally, the specific surface area (SSA) of TiO_2_ P25 and Ag–TiO_2_ NPs were measured to be 67 and 66 m^2^ g^−1^, respectively, showing that the surface area of pure TiO_2_ nanoparticles was practically unchanged by the introduction of Ag NPs. This indirectly proofs that the photocatalytic activity of Ag–TiO_2_ NPs under visible light will not be different from the one of pure TiO_2_ P25 NPs because of a change in surface area, as shown in the next section.

### Photocatalytic activity of Ag–TiO_2_ NPs towards CIP and NFX under visible light

After thoroughly characterizing the obtained material, the photocatalytic activity of Ag–TiO_2_ NPs towards the decomposition of two of the most used antibiotics belonging to the fluoroquinolone family, CIP and NFX, was investigated under visible light irradiation. The chemical structure of CIP and NFX can be found in Fig. S3 in ESI.†

**Fig. 5:**
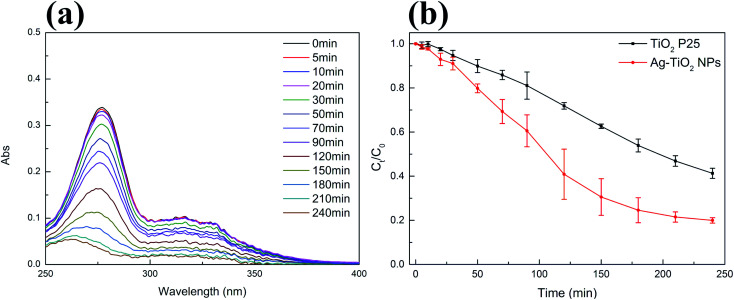
(a) Representative variations of the absorption spectrum of CIP (3 mg L^−1^, pH = 3) under visible light irradiation in the presence of Ag–TiO_2_ NPs (300 mg L^−1^); (b) time-dependent variation of the concentration of CIP solution upon exposure to visible light in the presence of TiO_2_ P25 (300 mg L^−1^, black squares) and Ag–TiO_2_ NPs (300 mg L^−1^, red circles); reported values are the mean of 3 replicates.

The photocatalytic performance of Ag–TiO_2_ NPs was initially evaluated by measuring the degradation of CIP in aqueous solution. Moreover, commercial TiO_2_ P25 was used as a reference. Before the photocatalytic experiment, photocatalysts were kept in contact with the antibiotic in dark conditions for 30 min to reach the adsorption–desorption equilibrium. The results showed, as expected, that there was no significant change in the pollutant concentration in dark conditions, which indicated that an adsorption–desorption equilibrium was reached after 30 minutes. Also, the concentration of CIP did not significantly change under visible light irradiation in the absence of the photocatalyst (Fig. S4(a) in ESI†), demonstrating the negligible photolysis of this molecule under visible light and the crucial role of the photocatalyst in the degradation of CIP in these conditions (Fig. 5(b)). Significant degradation of CIP, as it will be described more in detail below, occurs in the presence of both photocatalysts (TiO_2_ P25 and Ag–TiO_2_ NPs) under visible-light irradiation (Fig. 5(b)), as similarly reported in the literature.^48^ Nevertheless, from Fig. 5(b) it is already evident that the degradation of CIP is much faster in the presence of Ag–TiO_2_ NPs when compared to the TiO_2_ P25 reference. Fig. 5(a) shows that the UV-Vis maximum absorbance of CIP decreases with the increase of the irradiation time with visible light in the presence of Ag–TiO_2_ NPs. In particular, the concentration of CIP is decreased by 92% in the presence of Ag–TiO_2_ NPs after 240 min irradiation. Differently, within the same time, TiO_2_ P25 degraded only 67% of CIP, a value comparable to previously reported results.^15,49^ The high visible light photocatalytic activity of Ag–TiO_2_ NPs can be attributed to the optical properties of the system induced by the presence of the finely distributed Ag NPs on the surface of TiO_2_.

In order to investigate the photocatalytic activity of Ag–TiO_2_ NPs thoroughly, a kinetic study of the photodegradation process was performed. The obtained photocatalytic degradation curve was fitted by using a pseudo-first-order kinetic model as in eqn (1):^32^1
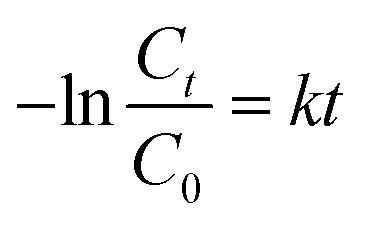
where *C*_*t*_ is the antibiotic concentration at time *t*, *C*_0_ is the antibiotic concentration at time zero and *k* is the degradation rate constant. The slope of the fitted curve gave the value of the degradation rate constant *k*. The linear relationship of −ln(*C*_*t*_/*C*_0_) *vs.* time is shown in Fig. S5(a),† and the values of the degradation rate constants are displayed in Table 1. The results indicate that the degradation rate constant for Ag–TiO_2_ NPs, in the case of CIP, is about 2.1 times higher than that for TiO_2_ P25.

**Table 1:** Photocatalytic reaction rate constant (*k*) values for the TiO_2_ P25 and Ag–TiO_2_ NPs

Catalyst	*k*_CIP_ (10^−3^ min^−1^)	*k*_NFX_ (10^−3^ min^−1^)
TiO_2_ P25	3.7	4.6
Ag–TiO_2_ NPs	7.6	7.9

**Fig. 6:**
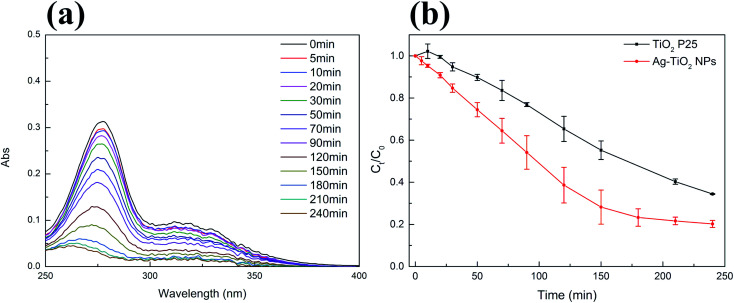
(a) Representative variations of the absorption spectrum of NFX (3 mg L^−1^, pH = 3) under visible light irradiation by using Ag–TiO_2_ NPs (300 mg L^−1^); (b) time-dependent concentration of NFX solution upon exposure to visible light in the presence of TiO_2_ P25 (300 mg L^−1^, black squares) and Ag–TiO_2_ NPs (300 mg L^−1^, red circles); reported values are the mean of 3 replicates.

We selected NFX as a second target compound to evaluate the visible light photocatalytic activity of Ag–TiO_2_ NPs. The concentration of NFX did also not significantly change under visible light irradiation in the absence of the photocatalyst (Fig. S4(b) in ESI†). As for CIP, the photodegradation of NFX using Ag–TiO_2_ NPs was studied by acquiring the absorption spectra of the NFX solution at different times of visible light irradiation in the presence of the photocatalyst, as shown in Fig. 6(a). In Fig. 6(b), the variation of NFX concentration over time shows that after 240 min of visible light exposure, the concentration of NFX decreased by 94% in the presence of Ag–TiO_2_ NPs and 76% in the presence of TiO_2_ P25. The pseudo-first-order-kinetic fitting (Fig. S5(b)† and Table 1) indicates that the degradation rate constant for Ag–TiO_2_ NPs is about 1.7 times higher than that for TiO_2_ P25. The degradation rate constants reported for similar catalysts, which were used in the photodegradation of CIP under similar irradiation conditions, are in the same range or even have lower values when compared with our findings.^32,50^ Higher degradation rate constants were also reported, but in this case the materials were either exhibiting different morphologies and/or Ag loading, or were tested under different irradiation conditions.^15,51,52^ Concerning NFX, a direct comparison of our catalysts performance with respect to the literature is rather difficult since the reported catalysts for the degradation of NFX possess different morphologies or are used in different experimental conditions.^53,54^ Based on our results, it can be concluded that the decoration of TiO_2_ surface with Ag NPs can significantly enhance the photocatalytic activity, under visible light irradiation, of the TiO_2_ P25 towards CIP and NFX.

Finally, we performed the recycling photocatalytic experiments to assess the photocatalytic performance of Ag–TiO_2_ NPs over different photocatalytic cycles. We chose CIP as a model pollutant for this experiment since it is the most commonly used antibiotic of its family and since we further studied the photocatalytic mechanism for this pharmaceutical in this work.

**Fig. 7:**
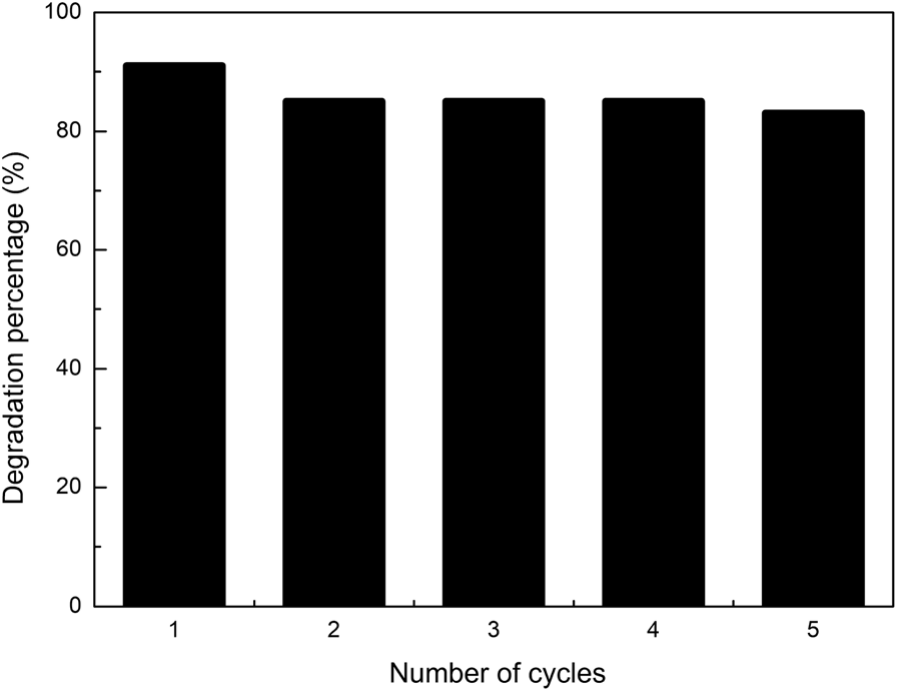
Five photocatalytic degradation reactions of fresh CIP solution (3 mg L^−1^, pH = 3) with reused (for up to 5 cycles) Ag–TiO_2_ NPs (300 mg L^−1^) under visible light irradiation.

The results for five photocatalytic cycles are reported in Fig. 7. Ag–TiO_2_ NPs were utilised in the photocatalytic degradation of CIP, recovered, washed once with 0.1 M HCl, twice with ultrapure water, and dried before being used for another photocatalytic experiment with a fresh CIP solution. This procedure was repeated five times.

As shown in Fig. 7, the visible-light photocatalytic efficiency of Ag–TiO_2_ NPs towards CIP only slightly decreased with the number of reutilisations. After five cycles, the amount of degraded CIP decreased only by 8%, namely from 91% to 83% of the initial CIP amount. The small decrease in the photocatalytic efficiency may be due to the loss of active sites caused by the adsorption on the surface of the catalyst of possibly unreacted CIP and/or its degradation products. The results obtained in this work are good and fit well with the previously published findings for similar catalysts, where comparable or even larger drops in catalyst efficiency were observed after cycling.^55–57^ Our results indicate that the prepared Ag–TiO_2_ NPs possess high stability and durability thus opening the path, after further studies, for a possible future industrial application.

### Photocatalytic mechanism of Ag–TiO_2_ NPs

Once the photocatalytic behaviour was thoroughly investigated, one of the main scopes of this work was to shed some light on the photocatalytic mechanism behind the enhanced activity of Ag–TiO_2_ NPs under visible light irradiation. CIP was selected as a model for the study of the photocatalytic degradation mechanism of Ag–TiO_2_ NPs towards the fluoroquinolone-based antibiotics since it is the most commonly used pharmaceutical of this family. Scavengers experiments were performed in order to determine which were the active species mainly involved in the photocatalytic degradation process.

**Fig. 8:**
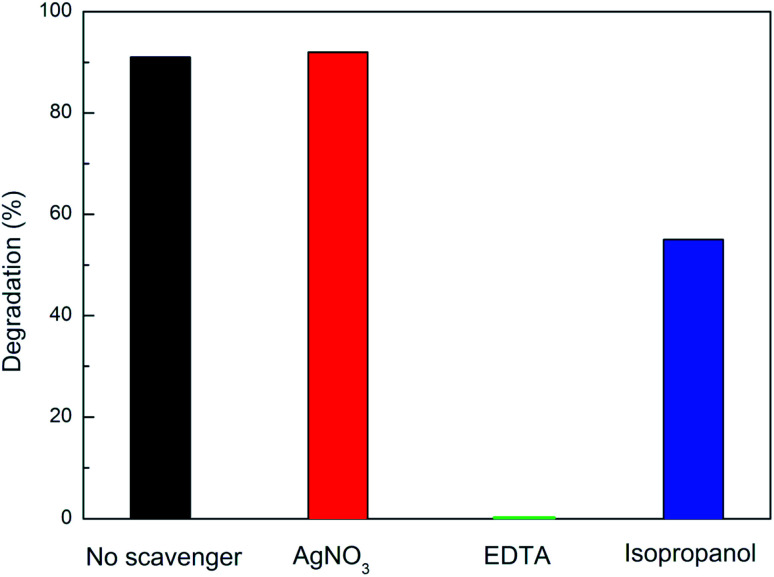
Visible-light photocatalytic degradation percentage of CIP (3 mg L^−1^, pH = 3) in the presence of only Ag–TiO_2_ NPs (300 mg L^−1^, in black) or of both Ag–TiO_2_ NPs (300 mg L^−1^) and the scavengers AgNO_3_ (in red), EDTA (in green) or isopropanol (in blue).

Fig. 8 shows that the photocatalytic degradation percentage of CIP by Ag–TiO_2_ NPs in the absence and the presence of different scavengers. When EDTA, a scavenger for holes (h^+^), was added into the reaction system, CIP degradation was significantly inhibited, and no CIP was degraded after 240 min irradiation. Another evident inhibition phenomenon for the photocatalytic reaction occurred in the presence of isopropanol, a scavenger for ˙OH radicals. Indeed, when isopropanol was added into the reaction system, the degradation efficiency of Ag–TiO_2_ NPs towards CIP decreased, resulting in 55% CIP degradation after 240 min irradiation. In contrast, in the case of the addition of AgNO_3_, a scavenger for electrons (e^−^), the photocatalytic degradation of CIP was nearly unchanged compared with the absence of scavengers. Our study indicates that CIP degradation is mainly caused by h^+^ and ˙OH, rather than e^−^ driven reactions taking place on the surface of the photocatalyst.^58^ Therefore, it can be concluded that h^+^ and ˙OH are the main active species of Ag–TiO_2_ NPs in aqueous solution under visible light irradiation.

**Scheme 2:**
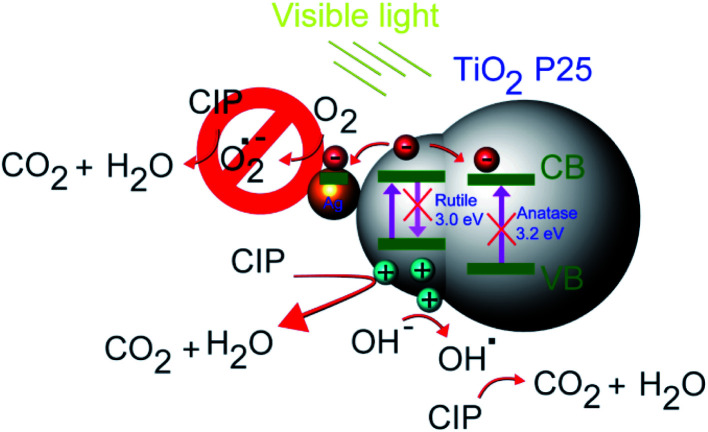
Photocatalytic mechanism scheme of Ag–TiO_2_ NPs under visible light.

This is in line with our previous findings for CIP degradation under visible light in the presence of other similar catalysts, such as ZnO.^59^ Based on the experimental results, a possible mechanism of visible light photocatalytic degradation of CIP in the presence of the Ag–TiO_2_ NPs is illustrated in Scheme 2. As well known, TiO_2_ P25 is a mixture of anatase and rutile phase in a ratio of about 80/20,^60,61^ respectively. D. C. Hurum *et al.*^62^ reported that rutile phase, due to its slightly lower bandgap in comparison to anatase, can be active under visible light irradation. This fact explains why TiO_2_ P25 can degrade CIP and NFX under visible light irradiation. However, the rutile phase is present in a much lower amount than anatase and, therefore, the photocatalytic activity is low, and the measured bandgap for TiO_2_ P25 resembles more the one of anatase. The presence of Ag NPs on the TiO_2_ P25 enhances this photocatalytic activity upon visible light irradiation due to a better electron–hole pairs' separation. When the Ag–TiO_2_ NPs are irradiated with visible light, the rutile phase of TiO_2_ will be activated. The photogenerated electrons can be then transferred to Ag NPs and to the anatase phase. In particular, Ag clusters deposited on the TiO_2_ surface will act as electrons traps avoiding the recombination of electrons and holes and, therefore, enhancing the photocatalytic activity of TiO_2_. This explains why the mechanism is mainly hole-driven. Holes and ˙OH radicals which are produced by the reaction between the holes and the water molecules adsorbed on the photocatalyst's surface play an important role in the degradation of CIP to CO_2_ and H_2_O. The degradation of CIP due to O_2_˙^−^ radicals is, instead, negligible based on our experiments with scavengers. Therefore, Ag contributes to the improvement of the photocatalytic activity of TiO_2_ under visible light, but the role played by the rutile phase in all the process seems to be essential and it was widely neglected in previous studies of TiO_2_ P25 based-systems under visible light irradiation.^37^ From the scavenger experiments, it can be estimated that ˙OH and h^+^ are the main active species in the photocatalytic degradation of CIP in the presence of Ag–TiO_2_ NPs in aqueous solution under visible light irradiation. In other reports about methylene blue degradation under visible light irradiation in the presence of Ag–TiO_2_ NPs, ˙OH radicals were found to be the main active species instead of holes and superoxide radicals.^55^ This stresses particularly the importance of the investigation of the photocatalytic mechanism for each specific pollutant/catalyst couple under certain selected conditions. However, one of the main results of this work concerns the importance of the rutile phase in the visible-light activation of TiO_2_ P25-based systems.

### Antibacterial activity of Ag–TiO_2_ NPs

**Fig. 9:**
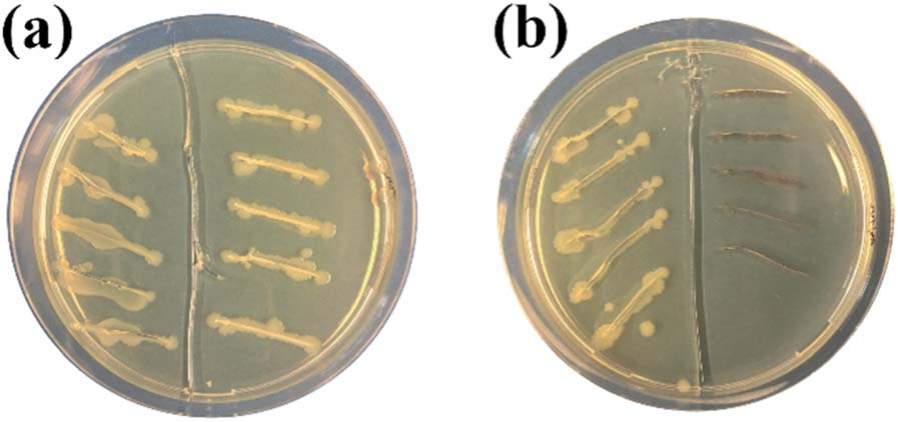
Modified streak method demonstrating the inhibitory bacteria growth potential of TiO_2_ (a) and Ag–TiO_2_ NPs (b) against *E. coli*: (a) *E. coli* in LB broth on the left part of the agar plate, TiO_2_ P25 and *E. coli* in LB broth on the right part of the agar plate; (b) *E. coli* in LB broth on the left part of the agar plate, Ag–TiO_2_ NPs and *E. coli* in LB broth on the right part of the agar plate.

Finally, the antibacterial properties of the Ag–TiO_2_ NPs towards *E. coli* bacteria were qualitatively tested in dark conditions. In Fig. 9, the results of a modified streaking method used to test the antibacterial activities of the two materials under study (TiO_2_ P25 and Ag–TiO_2_ NPs) are shown. In Fig. 9, *E. coli* in LB broth were added into each streak on the left part of the agar plates as a control. TiO_2_ P25 and *E. coli* in LB broth were added into each streak on the right part of the agar plate in Fig. 9(a). Ag–TiO_2_ NPs and *E. coli* in LB broth were added into each streak on the right part of the agar plate in Fig. 9(b). Plates were incubated at 37 °C for 24 h. Fig. 9(a) shows that the growth of *E. coli* is similar in each part (left and right). This indicates that TiO_2_ P25 cannot inhibit the growth of *E. coli*. Differently, in Fig. 9(b), there is no growth of *E. coli* on the right part of the plate compared to its left part. These findings mean that the Ag–TiO_2_ NPs can inhibit the growth of *E. coli* because of the presence of Ag NPs, which are known to have a robust antibacterial activity.^63^ Our previous research^64^ also showed that the antimicrobial activity of Ag NPs-based nanocomposites is closely related to the particle sizes of Ag NPs and their distribution on the surface of carrier particles. Smaller Ag NPs guarantee a better distribution on the carrier particles' surface, a greater solubility, and a higher concentration of antimicrobial active Ag^+^ ions released, resulting in an enhanced antimicrobial activity of the nanocomposite. Furthermore, small nanoparticles can penetrate bacterial cells and induce oxidative stress.^64^ From HRTEM images presented in this work, it is evident that Ag NPs are very finely distributed on the surface of TiO_2_ and that their sizes are in the range of the Ag NPs presented in our previous work.^64^ Therefore, the excellent *E. coli* growth inhibition ability of the Ag–TiO_2_ NPs can be entirely justified.

## Conclusions

In summary, Ag–TiO_2_ NPs are prepared by using a facile method. The resulting Ag–TiO_2_ NPs have an improved visible light-harvesting capacity and charge-separation efficiency compared with TiO_2_ P25 and thus possess excellent characteristics for photocatalysis under visible light irradiation. In these experimental conditions, Ag–TiO_2_ NPs show improved photocatalytic degradation properties towards CIP and NFX in comparison with those of commercial TiO_2_ P25. In particular, the degradation rate constants in the presence of Ag–TiO_2_ NPs increased by a factor of 2.1 and 1.7 for CIP and NFX respectively, compared with the values for commercial TiO_2_ P25. The recycling experiments showed that it is possible to reutilise the same Ag–TiO_2_ NPs batch for five consecutive CIP photodegradation reactions with a drop in efficiency limited to 8%. Moreover, it was demonstrated that ˙OH and h^+^ play a major role in the photocatalytic degradation of CIP under visible light irradiation in the presence of Ag–TiO_2_ NPs and that the rutile phase in the TiO_2_ P25-based systems should be not neglected in the activation mechanism of TiO_2_ under visible light. Furthermore, Ag–TiO_2_ NPs can inhibit the growth of *E. coli* significantly.

We believe that our manuscript provides important clarifications about the possible photocatalytic mechanism under visible light irradiation for Ag NPs decorated TiO_2_ P25 systems. Altogether, the prepared Ag–TiO_2_ NPs are a viable alternative for the degradation of fluoroquinolone-based antibiotics under visible light irradiation and, at the same time, for the inactivation of bacteria in water. It is foreseen that the Ag–TiO_2_ NPs will find practical utilisation and application in water treatment, especially in that of hospital wastewater, where the removal of pharmaceuticals and the disinfection from bacteria is a desirable final aim.

## Conflicts of interest

The authors declare no conflict of interest.

## Supplementary Material

RA-011-D0RA10403E-s001

## References

[cit1] Schwarzenbach P., Egli T., Hofstetter T. B., Von Gunten U., Wehrli B. (2010). Global water pollution and human health. Annu. Rev. Environ. Resour..

[cit2] Bhuiyan A. B., Mokhtar M., Toriman M. E., Gasim M. B., Ta G. C., Elfithri R., Razman M. R. (2013). The environmental risk and water pollution: A review from the river basins around the world. Am.-Eurasian J. Sustain. Agric..

[cit3] Durán-Álvarez J. C., Avella E., Ramírez-Zamora R. M., Zanella R. (2016). Photocatalytic degradation of ciprofloxacin using mono-(Au, Ag and Cu) and bi- (Au-Ag and Au-Cu) metallic nanoparticles supported on TiO_2_ under UV-C and simulated sunlight. Catal. Today.

[cit4] Ali T., Ahmed A., Alam U., Uddin I., Tripathi P., Muneer M. (2018). Enhanced photocatalytic and antibacterial activities of Ag-doped TiO_2_ nanoparticles under visible light. Mater. Chem. Phys..

[cit5] Carlsson C., Johansson A., Alvan G., Bergman K., Ku T. (2006). Are pharmaceuticals potent environmental pollutants? Part I: Environmental risk assessments of selected active pharmaceutical ingredients. Sci. Total Environ..

[cit6] Hough W. L., Rogers R. D. (2007). Ionic liquids then and now: from solvents to materials to active pharmaceutical ingredients. Bull. Chem. Soc. Jpn..

[cit7] Maia A. S., Ribeiro A. R., Amorim C. L., Barreiro J. C., Cass Q. B., Castro P. M. L., Tiritan M. E. (2014). Degradation of fluoroquinolone antibiotics and identification of metabolites/transformation products by liquid chromatography-tandem mass spectrometry. J. Chromatogr. A.

[cit8] Teixeira S., Gurke R., Eckert H., Kühn K., Fauler J., Cuniberti G. (2016). Photocatalytic degradation of pharmaceuticals present in conventional treated wastewater by nanoparticle suspensions. J. Environ. Chem. Eng..

[cit9] Daughton C. (2002). Environmental stewardship and drugs as pollutants. Lancet.

[cit10] Xu W., Zhang G., Li X., Zou S., Li P., Hu Z., Li J. (2007). Occurrence and elimination of antibiotics at four sewage treatment plants in the Pearl River Delta (PRD), South China. Water Res..

[cit11] Shi L., Zhou X. F., Zhang Y. L., Gu G. W. (2009). Simultaneous determination of 8 fluoroquinolone antibiotics in sewage treatment plants by solid-phase extraction and liquid chromatography with fluorescence detection. Water Sci. Technol..

[cit12] Larsson D. G. J., de Pedro C., Paxeus N. (2007). Effluent from drug manufactures contains extremely high levels of pharmaceuticals. J. Hazard. Mater..

[cit13] Li D., Yang M., Hu J., Zhang Y., Chang H., Jin F. (2008). Determination of penicillin G and its degradation products in a penicillin production wastewater treatment plant and the receiving river. Water Res..

[cit14] Mahdi-Ahmed M., Chiron S. (2014). Ciprofloxacin oxidation by UV-C activated peroxymonosulfate in wastewater. J. Hazard. Mater..

[cit15] Jiang Z., Zhu J., Liu D., Wei W., Xie J., Chen M. (2014). In situ synthesis of bimetallic Ag/Pt loaded single-crystalline anatase TiO_2_ hollow nano-hemispheres and their improved photocatalytic properties. CrystEngComm.

[cit16] Pearce C. I., Lloyd J. R., Guthrie J. T. (2003). The removal of colour from textile wastewater using whole bacterial cells: A review. Dyes Pigm..

[cit17] Van Der Bruggen B., Vandecasteele C. (2003). Removal of pollutants from surface water and groundwater by nanofiltration: Overview of possible applications in the drinking water industry. Environ. Pollut..

[cit18] Hollender J., Zimmermann S. G., Koepke S., Krauss M., McArdell C. S., Ort C., Singer H., Von Gunten U., Siegrist H. (2012). Elimination of organic micropollutants in a municipal wastewater treatment plant upgraded with a full scale post-ozonation followed by sand filtration. Environ. Sci. Technol..

[cit19] Hu C. Y., Lo S. L., Kuan W. H. (2003). Effects of co-existing anions on fluoride removal in electrocoagulation (EC) process using aluminum electrodes. Water Res..

[cit20] Iram M., Guo C., Guan Y., Ishfaq A., Liu H. (2010). Adsorption and magnetic removal of neutral red dye from aqueous solution using Fe_3_O_4_ hollow nanospheres. J. Hazard. Mater..

[cit21] Saud P. S., Pant B., Alam A. M., Ghouri Z. K., Park M., Kim H. Y. (2015). Carbon quantum dots anchored TiO_2_ nanofibers: Effective photocatalyst for waste water treatment. Ceram. Int..

[cit22] Friedler E., Gilboa Y. (2010). Performance of UV disinfection and the microbial quality of greywater effluent along a reuse system for toilet flushing. Sci. Total Environ..

[cit23] Ibhadon A. O., Fitzpatrick P. (2013). Heterogeneous photocatalysis: recent advances and applications. Catalysts.

[cit24] Ma Y., Wang X., Jia Y., Chen X., Han H., Li C. (2014). Titanium dioxide-based nanomaterials for photocatalytic fuel generations. Chem. Rev..

[cit25] Fujishima A., Honda K. (1972). Electrochemical photolysis of water at a semiconductor electrode. Nature.

[cit26] Jia C., Zhang X., Matras-Postolek K., Huang B., Yang P. (2018). Z-scheme reduced graphene oxide/TiO_2_-Bronze/W_18_O_49_ ternary heterostructure towards efficient full solar-spectrum photocatalysis. Carbon.

[cit27] Nasr M., Soussan L., Viter R., Eid C., Habchi R., Miele P., Bechelany M. (2018). High photodegradation and antibacterial activity of BN-Ag/TiO_2_ composite nanofibers under visible light. New J. Chem..

[cit28] Shang M., Hou H., Gao F., Wang L., Yang W. (2017). Mesoporous Ag@TiO_2_ nanofibers and their photocatalytic activity for hydrogen evolution. RSC Adv..

[cit29] Yar A., Haspulat B., Üstün T., Eskizeybek V., Avci A., Kamiş H., Achour S. (2017). Electrospun TiO_2_/ZnO/PAN hybrid nanofiber membranes with efficient photocatalytic activity. RSC Adv..

[cit30] Keller V., Bernhardt P., Garin F. (2003). Photocatalytic oxidation of butyl acetate in vapor phase on TiO_2_, Pt/TiO_2_ and WO_3_/TiO_2_ catalysts. J. Catal..

[cit31] Pal B., Sharon M., Nogami G. (1999). Preparation and characterisation of TiO_2_/Fe_2_O_3_ binary mixed oxides and its photocatalytic properties. Mater. Chem. Phys..

[cit32] Martins P., Kappert S., Le H. N., Sebastian V., Kühn K., Alves M., Pereira L., Cuniberti G., Melle-Franco M., Lanceros-Méndez S. (2020). Enhanced photocatalytic activity of au/TiO_2_ nanoparticles against ciprofloxacin. Catalysts.

[cit33] Yao Y. C., Dai X. R., Hu X. Y., Huang S. Z., Jin Z. (2016). Synthesis of Ag-decorated porous TiO_2_ nanowires through a sunlight induced reduction method and its enhanced photocatalytic activity. Appl. Surf. Sci..

[cit34] Scott T., Zhao H., Deng W., Feng X., Li Y. (2019). Photocatalytic degradation of phenol in water under simulated sunlight by an ultrathin MgO coated Ag/TiO_2_ nanocomposite. Chemosphere.

[cit35] Ko S., Banerjee C. K., Sankar J. (2011). Photochemical synthesis and photocatalytic activity in simulated solar light of nanosized Ag doped TiO_2_ nanoparticle composite. Composites, Part B.

[cit36] Zhang L., Ji Y., Wu D., Du S., Zhang S., Zhou S. (2017). Controlled synthesis of Ag/TiO_2_ nanotube arrays composites with different Ag loading and their enhanced photoelectrochemical and photocatalytic performance. J. Nanosci. Nanotechnol..

[cit37] Méndez-Medrano M. G., Kowalska E., Lehoux A., Herissan A., Ohtani B., Bahena D., Briois V., Colbeau-Justin C., Rodríguez-López J. L., Remita H. (2016). Surface modification of TiO_2_ with Ag nanoparticles and CuO nanoclusters for application in photocatalysis. J. Phys. Chem. C.

[cit38] Chen K., Feng X., Tian H., Li Y., Xie K., Hu R., Cai Y., Gu H. (2014). Silver-decorated titanium dioxide nanotube arrays with improved photocatalytic activity for visible light irradiation. J. Mater. Res..

[cit39] Roca Jalil M. E., Baschini M., Sapag K. (2015). Influence of pH and antibiotic solubility on the removal of ciprofloxacin from aqueous media using montmorillonite. Appl. Clay Sci..

[cit40] Eythorsdottir A., Omarsdottir S., Einarsson H. (2016). Antimicrobial activity of marine bacterial symbionts retrieved from shallow water hydrothermal vents. Mar. Biotechnol..

[cit41] Gurunathan S., Umashankar V., Murugesan S., Dhamotharan R. (2014). 16s rDNA based molecular identification of Bacteriocin-like inhibitory substance (BLIS/BIS) producing indigenous phytopathogenic bacteria isolated from various diseased plant materials. Int. J. Curr. Sci..

[cit42] Duan Y., Zhang M., Wang L., Wang F., Yang L., Li X., Wang C. (2017). Plasmonic Ag-TiO_2_-x nanocomposites for the photocatalytic removal of NO under visible light with high selectivity: The role of oxygen vacancies. Appl. Catal., B.

[cit43] Kamat P. V. (2002). Photophysical, photochemical and photocatalytic aspects of metal nanoparticles. J. Phys. Chem. B.

[cit44] Albiter E., Valenzuela M. A., Alfaro S., Valverde-Aguilar G., Martínez-Pallares F. M. (2015). Photocatalytic deposition of Ag nanoparticles on TiO_2_: Metal precursor effect on the structural and photoactivity properties. J. Saudi Chem. Soc..

[cit45] Koo Y., Littlejohn G., Collins B., Yun Y., Shanov V. N., Schulz M., Pai D., Sankar J. (2014). Synthesis and characterization of Ag-TiO_2_-CNT nanoparticle composites with high photocatalytic activity under artificial light. Composites, Part B.

[cit46] Lim S. P., Pandikumar A., Huang N. M., Lim H. N. (2014). Enhanced photovoltaic performance of silver@titania plasmonic photoanode in dye-sensitized solar cells. RSC Adv..

[cit47] Wei N., Cui H., Song Q., Zhang L., Song X., Wang K., Zhang Y., Li J., Wen J., Tian J. (2016). Ag_2_O nanoparticle/TiO_2_ nanobelt heterostructures with remarkable photo-response and photocatalytic properties under UV, visible and near-infrared irradiation. Appl. Catal., B.

[cit48] Jiang Z., Wei W., Mao D., Chen C., Shi Y., Lv X., Xie J. (2015). Silver-loaded nitrogen-doped yolk-shell mesoporous TiO_2_ hollow microspheres with enhanced visible light photocatalytic activity. Nanoscale.

[cit49] Paul T., Miller P. L., Strathmann T. J. (2007). Visible-light-mediated TiO_2_ photocatalysis of fluoroquinolone antibacterial agents. Environ. Sci. Technol..

[cit50] Tang Y., Sun H., Shang Y., Zeng S., Qin Z., Yin S., Li J., Liang S., Lu G., Liu Z. (2019). Spiky nanohybrids of titanium dioxide/gold nanoparticles for enhanced photocatalytic degradation and anti-bacterial property. J. Colloid Interface Sci..

[cit51] Liu Z., Ma Z. (2019). Ag-SrTiO_3_/TiO_2_ composite nanostructures with enhanced photocatalytic activity. Mater. Res. Bull..

[cit52] Durán-Álvarez J. C., Avella E., Ramírez-Zamora R. M., Zanella R. (2016). Photocatalytic degradation of ciprofloxacin using mono-(Au, Ag and Cu) and bi-(Au-Ag and Au-Cu) metallic nanoparticles supported on TiO_2_ under UV-C and simulated sunlight. Catal. Today.

[cit53] Salazar H., Martins P. M., Santos B., Fernandes M. M., Reizabal A., Sebastián V., Botelho G., Tavares C. J., Vilas-Vilela J. L., Lanceros-Mendez S. (2020). Photocatalytic and antimicrobial multifunctional nanocomposite membranes for emerging pollutants water treatment applications. Chemosphere.

[cit54] Lin Z., Lu Y., Huang J. (2019). A hierarchical Ag_2_O-nanoparticle/TiO_2_-nanotube composite derived from natural cellulose substance with enhanced photocatalytic performance. Cellulose.

[cit55] Ali T., Ahmed A., Alam U., Uddin I., Tripathi P., Muneer M. (2018). Enhanced photocatalytic and antibacterial activities of Ag-doped TiO_2_ nanoparticles under visible light. Mater. Chem. Phys..

[cit56] Kaur A., Anderson W. A., Tanvir S., Kansal S. K. (2019). Solar light active silver/iron oxide/zinc oxide heterostructure for photodegradation of ciprofloxacin, transformation products and antibacterial activity. J. Colloid Interface Sci..

[cit57] Xu J., Liu Y., Zhao Y. (2020). Effect of Ag loading position on the photocatalytic performance of TiO_2_ nanocolumn arrays. Beilstein J. Nanotechnol..

[cit58] Li W., Seal S., Megan E., Ramsdell J., Scammon K., Lelong G., Lachal L., Richardson K. A. (2003). Physical and optical properties of sol-gel nano-silver doped silica film on glass substrate as a function of heat-treatment temperature. J. Appl. Phys..

[cit59] Ulyankina A., Molodtsova T., Gorshenkov M., Leontyev I., Zhigunov D., Konstantinova E., Lastovina T., Tolasz J., Henych J., Licciardello N., Cuniberti G., Smirnova N. (2021). Photocatalytic degradation of ciprofloxacin in water at nano-ZnO prepared by pulse alternating current electrochemical synthesis. J. Water Process. Eng..

[cit60] Tobaldi D. M., Pullar R. C., Seabra M. P., Labrincha J. A. (2014). Fully quantitative X-ray characterisation of Evonik Aeroxide TiO_2_ P25®. Mater. Lett..

[cit61] Amano F., Nakata M., Yamamoto A., Tanaka T. (2016). Rutile titanium dioxide prepared by hydrogen reduction of Degussa P25 for highly efficient photocatalytic hydrogen evolution. Catal. Sci. Technol..

[cit62] Hurum D. C., Agrios A. G., Gray K. A., Rajh T., Thurnauer M. C. (2003). Explaining the enhanced photocatalytic activity of Degussa P25 mixed-phase TiO_2_ using EPR. J. Phys. Chem. B.

[cit63] Gong P., Li H., He X., Wang K., Hu J., Tan W., Zhang S., Yang X. (2007). Preparation and antibacterial
activity of Fe_3_O_4_@Ag nanoparticles. Nanotechnology.

[cit64] Svoboda L., Bednář J., Dvorský R., Rybková Z., Malachová K., Henych J., Matýsek D., Němečková Z. (2020). Novel synthesis of Ag@AgCl/ZnO by different radiation sources including radioactive isotope ^60^Co: Physicochemical and antimicrobial study. Appl. Surf. Sci..

