# Supplementary material for: Enhanced visible-light photodegradation of fluoroquinolone-based antibiotics and E. coli growth inhibition using Ag–TiO2 nanoparticles
Source: RSC Adv. 2021 Apr 13;11(23):13980–91. doi: 10.1039/d0ra10403e (PMC8697706; doi:10.1039/d0ra10403e)
Supplement: RA-011-D0RA10403E-s001 [file RA-011-D0RA10403E-s001.pdf]

Electronic Supplementary Material (ESI) for RSC Advances.
This journal is © The Royal Society of Chemistry 2021

Supporting Information

Enhanced visible-light photodegradation of fluoroquinolone-based antibiotics and *E. coli* growth inhibition using Ag-TiO_2 nanoparticles

Jiao Wang [a], Ladislav Svoboda [b,c], Zuzana Něměčková [d], Massimo Sgarzi [a], Jiří Henych [d], Nadia Licciardello [a,‡*], Gianaurelio Cuniberti [a*]

[a] Institute for Materials Science and Max Bergmann Centre of Biomaterials, TU Dresden, 01062, Dresden, Germany

[b] IT4Innovations, VŠB – Technical University of Ostrava, 17. listopadu 15/2172, 708 33 Ostrava, Czech Republic

[c] Nanotechnology Centre, CEET, VSB-Technical University of Ostrava, 17. listopadu 15/2172, 708 33 Ostrava, Czech Republic

[d] Institute of Inorganic Chemistry, Czech Academy of Sciences, Husinec-Řež 1001, 250 68 Řež, Czech Republic

[‡] Current address: International Iberian Nanotechnology Laboratory, Avenida Mestre José Veiga s/n, 4715-330 Braga, Portugal

[*] Corresponding authors:
Nadia Licciardello, E-mail: nadia.licciardello@tu-dresden.de and nadia.licciardello@inl.int;
Gianaurelio Cuniberti, E-mail: gianaurelio.cuniberti@tu-dresden.de

The ATR-FTIR spectra of TiO_2 P25 and Ag-TiO_2 NPs are shown in Figure S1. The band between 500 cm^{-1} and 700 cm^{-1} visible in both spectra of TiO_2 P25 and Ag-TiO_2 NPs corresponds to the Ti-O-Ti stretching vibrations[1]. The broad band at 3380 cm^{-1} corresponds to the O-H stretching vibrations due to adsorption of water molecules from the moisture[1]. The FTIR spectrum of Ag-TiO_2 NPs resembles the one of TiO_2 NPs very much. However, the presence of Ag NPs in the Ag-TiO_2 NPs sample is hard to be

shown by FTIR spectroscopy since their concentration is low compared to the TiO_2 NPs.

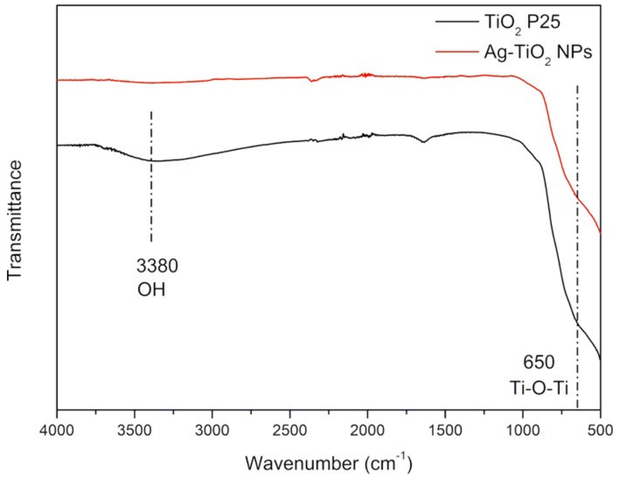

Figure S1. ATR-FTIR spectra of TiO_2 P25 and Ag-TiO_2 NPs

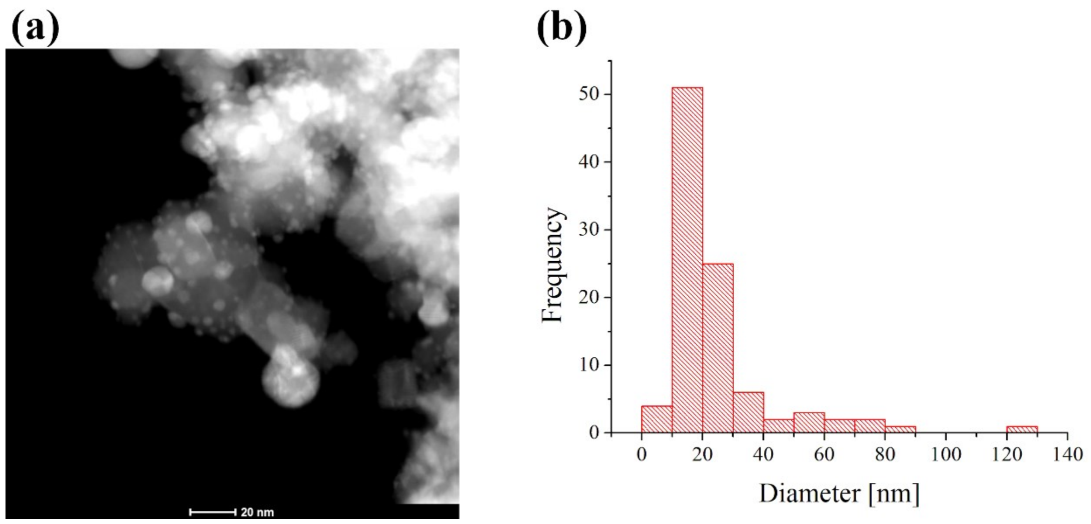

Figure S2. (a) STEM-HAADF micrograph of Ag/TiO_2 NPs; (b) Size distribution graph for Ag NPs obtained from Figure S2(a).

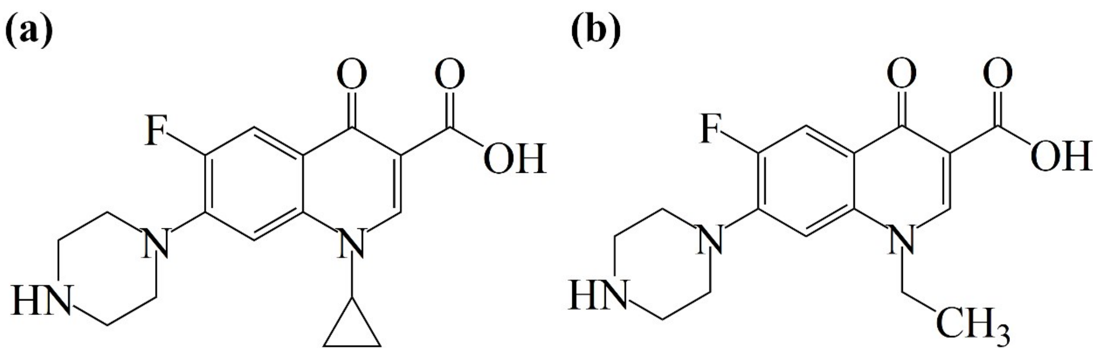

Figure S3. (a) Chemical structure of CIP; (b) Chemical structure of NFX.

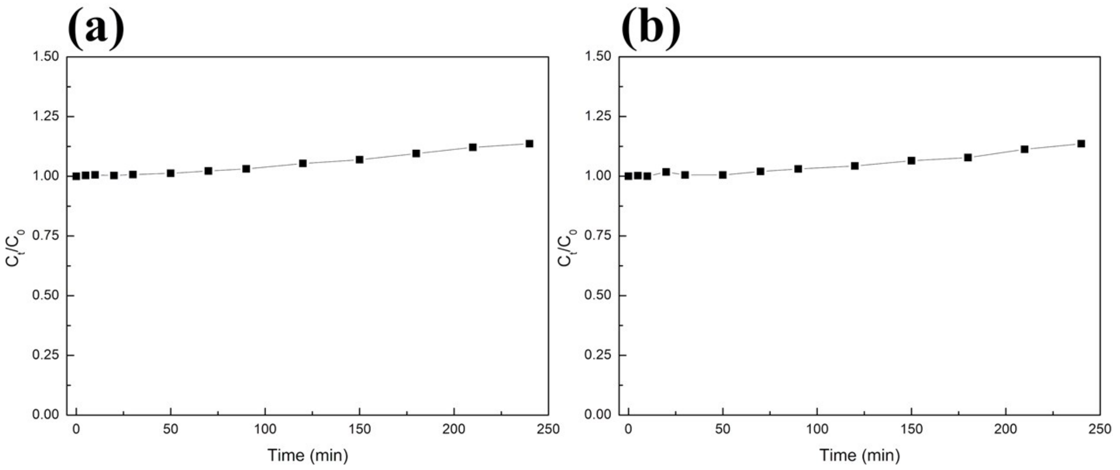

Figure S4. (a) Time-dependent variation of the concentration of CIP solution (3 mg/L, pH = 3) upon exposure to visible light; (b) Time-dependent variation of the concentration of NFX solution (3 mg/L, pH = 3) upon exposure to visible light.

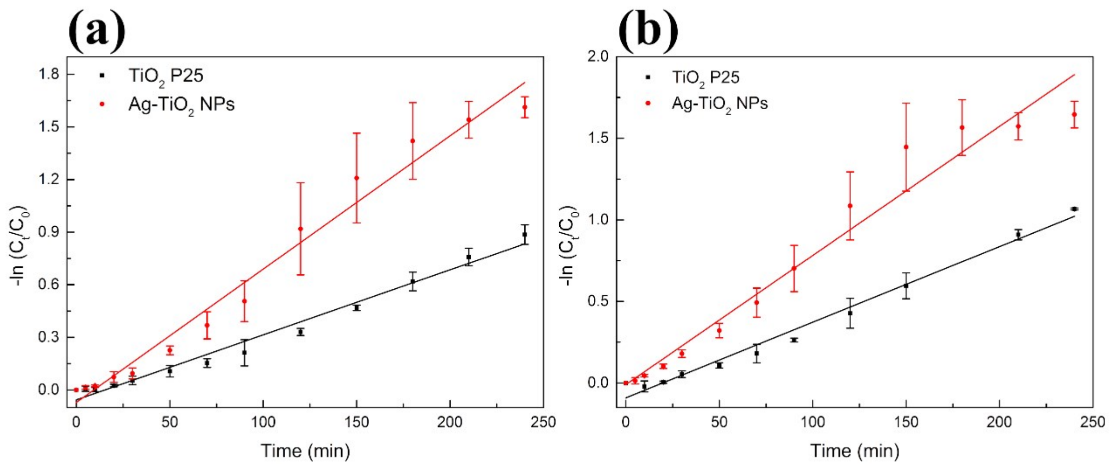

Figure S5. Pseudo-first order kinetic fitting for: (a) the CIP solution (3 mg/L, pH = 3) upon exposure to visible light in the presence of TiO_2 P25 (300 mg/L) and Ag-TiO_2 NPs (300 mg/L); (b) the NFX solution (3 mg/L, pH = 3) upon exposure to visible light in the presence of TiO_2 P25 (300 mg/L) and Ag-TiO_2 NPs (300 mg/L).

References

1. E. H. Alsharaeh, T. Bora, A. Soliman, F. Ahmed, G. Bharath, M. G. Ghoniem, K. M.

Abu-Salah and J. Dutta, Sol-gel-assisted microwave-derived synthesis of anatase Ag/TiO_2/GO Nanohybrids toward efficient visible light phenol degradation, *Catalysts*, 2017, **7**, 133.
